# 195. Duration of Therapy for Streptococcal Bacteremia

**DOI:** 10.1093/ofid/ofab466.397

**Published:** 2021-12-04

**Authors:** John M Boulos, Valeria Fabre, Kate Dzintars, Kate Dzintars, George Jones, Sara E Cosgrove, Sara E Cosgrove, Fidelia Bernice, Pranita Tamma, Pranita Tamma

**Affiliations:** 1 The Johns Hopkins Hospital, Durham, North Carolina; 2 Johns Hopkins University, Baltimore, Maryland; 3 Johns Hopkins University School of Medicine, Baltimore, Maryland; 4 Johns Hopkins, Baltimore, MD

## Abstract

**Background:**

Shorter durations have shown similar clinical outcomes as longer durations for uncomplicated (source-controlled) Gram-negative bloodstream infections (BSI). There is limited data on the outcomes of patients with non-pneumococcal streptococcal BSI receiving shorter durations of therapy compared to usual durations.

**Methods:**

This was a retrospective, multicenter study of adults hospitalized between January 2018 and March 2019 with ≥ 1 blood culture positive for Streptococcus spp. Exposed patients were those who received ≤ 10 days of antibiotics (i.e., short course therapy) and unexposed patients were those who received 11-21 days of antibiotics (i.e., prolonged course therapy). Patients were excluded if they had *S. pneumoniae* BSI, suspected contamination, did not receive or complete therapy, or treated for > 21 days. The primary outcome was a composite of recurrent bacteremia with the same pathogen, hospital readmission, or all-cause mortality, all within 30 days from completing therapy. The odds of achieving the primary outcome was compared between exposed and unexposed patients using multivariable logistic regression analysis.

**Results:**

A total of 176 patients met eligibility criteria. 35 (20%) received a short course (median 8 days) and 141 (80%) received a prolonged course (median 15 days) of antibiotic therapy. Baseline characteristics were similar between short and long course groups. The most common pathogens were viridans group streptococci (22%) and *S. agalactiae* (23%). The most common BSI source was skin and soft tissue infection (SSTI) (40%). The primary outcome occurred in 26% (9/35) and 23% (33/141) of patients in the short course and prolonged course groups, respectively (p = 0.774). The proportion of patients in the short course and prolonged course groups who experienced recurrent BSI, hospital readmission, or all-cause mortality were also non-significant. After adjusting for receipt of an infectious diseases consult, Pitt bacteremia score, and SSTI source, the adjusted odds of meeting the composite outcome remained unchanged (aOR 1.41, 95% CI 0.55 – 3.61, p = 0.466).

Table 1. Cohort Characteristics

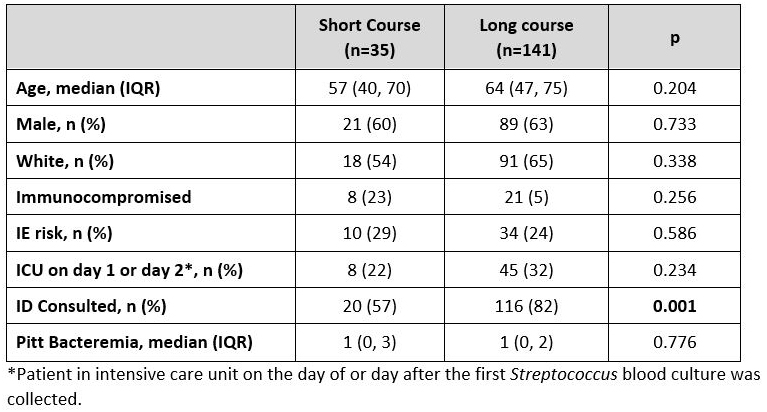

Table 2. Source/Microbiology

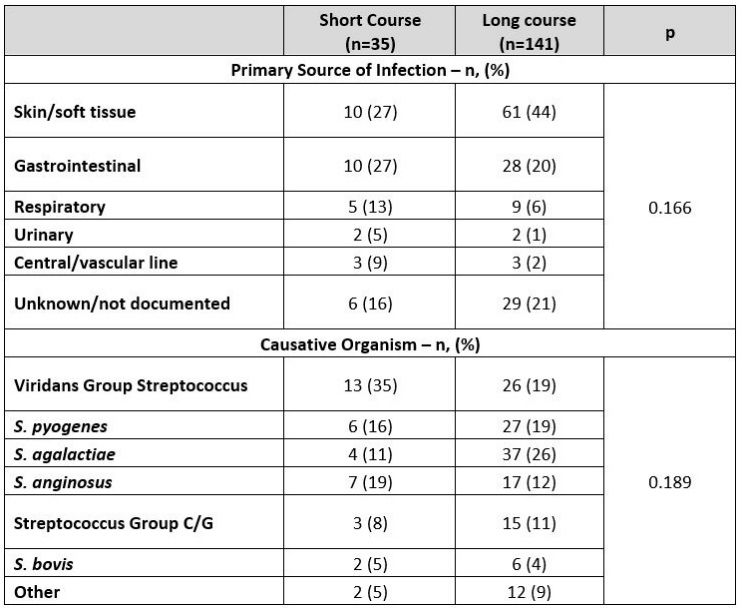

Table 3. Outcomes

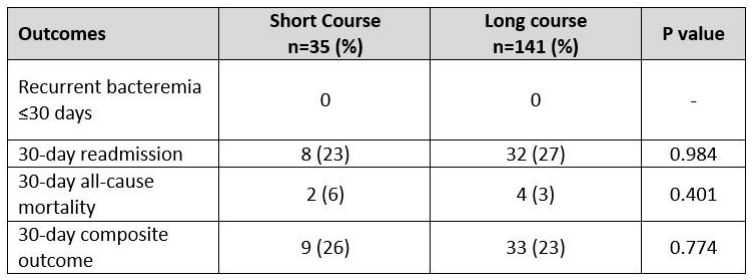

**Conclusion:**

Approximately a week of antibiotic therapy may be associated with similar clinical outcomes as longer antibiotics courses in patients with uncomplicated streptococcal BSI.

**Disclosures:**

**Kate Dzintars, PharmD**, Nothing to disclose **Sara E. Cosgrove, MD, MS**, Basilea (Individual(s) Involved: Self): Consultant **Pranita Tamma, MD, MHS**, Nothing to disclose

